# Home Language Activities and Language Ability Between Chinese Preschool Children with Cochlear Implants and Children with Normal Hearing

**DOI:** 10.3390/audiolres16010018

**Published:** 2026-01-28

**Authors:** Meilin He, Inho Chung

**Affiliations:** 1 Doctoral Program in Disability Sciences, Graduate School of Comprehensive Human Sciences, University of Tsukuba, Tsukuba 305-8577, Japan; 2 Institute of Human Sciences, University of Tsukuba, Tsukuba 305-8577, Japan

**Keywords:** hearing-impaired, preschool children with cochlear implants, language activities, language ability

## Abstract

This study explored the relationship between different home language activities and language ability in Chinese preschoolers with cochlear implants (CIs) (mean age = 4.50, range = 3–5), comparing them with normally hearing (NH) peers (mean age = 4.66, range = 3–5). Correlation and regression analyses revealed distinct predictive patterns between the two groups. In the CI group, although family literacy activities such as shared reading were associated with language skills, daily communication activities (e.g., conversational interactions) had a more significant predictive effect on language outcomes, even after controlling for key demographic variables. Conversely, for NH preschool children, family literacy activities showed a clearer independent association with language development. This study offers clearer insights for home-based rehabilitation practices among CI preschool children, suggesting that interventions should prioritize high-quality daily communication (e.g., open-ended questioning, extended dialog, contextualized interactions) rather than over-reliance on structured literacy activities. It also indicates that intervention models designed for NH preschool children cannot be simply applied.

## 1. Introduction

Language acquisition in early childhood serves as the foundation for cognitive development and social skills [1]. However, hearing impairment can disrupt this critical process [2]. In recent years, with the widespread adoption of newborn hearing screening and cochlear implant (CI) surgery in China, the number of CI users has grown rapidly, and the age of implantation has shown a trend toward younger children [3,4,5,6].

CIs improve auditory input, however, studies consistently report significant individual differences in language development outcomes among users. These variations are associated not only with factors such as age at implantation and the surgical procedure itself, but also closely relate to the post-operative rehabilitation environment, particularly whether the family can provide sufficient and high-quality language input and interaction (e.g., parental involvement, home training) [7,8,9,10,11,12]. Moreover, even with CI or CIs, the ability to perceive sounds in noisy environments differs from that in quiet settings [13].

As mentioned above, the use of CI does not guarantee that a child will develop listening, speaking, reading, and writing abilities identical to those of NH children. However, research has shown that language acquisition significantly influences cognitive development [14] and that early language input plays a crucial role in children’s linguistic and cognitive growth [15]. Language development during preschool years has been shown to have a significant impact on subsequent academic achievement and social skill development [16]. These findings highlight the need for enhanced and individualized language development support for hearing-impaired preschool children with CIs [8].

Research has shown that the period between 0 and 6 years of age is considered the “golden period for rehabilitation” due to the high levels of neuroplasticity during this time, because this neural normalization is strongly correlated with key behavioral outcomes. It typically translates to superior auditory perceptual skills, such as the ability to discriminate fine phonetic contrasts, recognize speech in noisy environments, and localize sounds more accurately [17]. For example, studies using electroencephalography (EEG), a method to record electrical activity from the brain, have shown that children who receive auditory intervention (cochlear implant) before the age of 3 years can develop auditory cortical function close to that of NH preschool children. However, the effectiveness of the intervention significantly decreased when performed after the age of 7 [18]. Ultimately, these enhanced auditory processing capabilities form a more robust neural foundation for the development of spoken language comprehension and production [17]. In practice, this intervention typically involves a combination of advanced sensory devices (e.g., hearing aids followed by cochlear implantation) paired with consistent, auditory-rich language exposure and professional auditory training. In addition, research by Nicholas and Geers (2007) showed that children who receive CIs early (especially before the age of 2 years) show language development similar to that of NH preschool children [19]. Furthermore, longitudinal studies have confirmed that children who receive CIs before the age of 2 have significantly better language abilities at the age of 6 compared to those who receive implants later [20]. This underscores the importance of early intervention for optimal language and auditory development.

Since the implementation of newborn hearing screening and cochlear implantation, numerous studies have been conducted on the effects of CIs on language development post-implantation. For example, research has focused on the effects of CIs after language training. However, it has been found that the success of CI usage in young children largely depends on the active involvement of caregivers, which leads to improvements in speech production and clarity [21]. Caregivers’ language behaviors also play a crucial role in enhancing the language abilities of children with CIs [22], and experiences with reading and writing at home have been shown to influence language development [23]. Research has shown that the home environment is often referred to as the “first classroom” [24], and the language environment at home is strongly correlated with vocabulary development, which in turn is closely linked to academic performance [25]. Additionally, it has been pointed out that in China, the language development of Hearing-impaired preschool children with CIs is similarly influenced by their parents’ educational background and home environment [26]. Several studies have been conducted abroad on the use of language input at home for children with hearing impairment. For example, research has reported that in households with hearing-impaired infants and toddlers, interactive language input (e.g., responsive dialog, joint attention) is positively correlated with language development scores, whereas one-sided directive language is ineffective [27]. Moreover, studies have indicated that in families with children using CIs, maternal sensitivity (prompt responses to the child’s cues) and cognitive stimulation (such as shared reading and questioning) can significantly predict language development [28]. Parent-led language interventions, such as dialog skill training and shared reading strategies, have been shown to significantly improve children’s language abilities [29]. Furthermore, numerous studies have demonstrated that various language activities are beneficial to children’s language development. For example, shared reading of picture books [30,31,32,33], cognitively challenging conversations [30,34,35,36], direct vocabulary instruction [37,38], and play [39,40,41] have all been found to positively impact children’s language abilities and early reading skills. For CI preschool children, the home environment is particularly significant because, at a young age, they spend a substantial amount of time with their families. In other words, the diverse language activities within the family environment exert a crucial influence on children’s language development through active, interactive engagement. Theories on the definition of language activities vary both domestically and internationally [42,43,44,45,46,47,48,49,50,51]. In summary, language activities broadly refer to all interactions or processes centered on language use, aimed at achieving communication, learning, or expression. Their forms extensively encompass listening, speaking, reading, writing, and language-based thinking. Specifically within the family environment, this includes all intentional or unintentional interactions centered on language, such as daily conversations, parent–child reading, and communication during play [42,45].

Family language activities, such as shared reading and play, have been extensively demonstrated to effectively promote language and literacy development in children, including those with cochlear implants (CI) [32,33,39,45,52,53]. However, there remains a significant gap in research within this field in China. First, existing literature predominantly focuses on clinical medical interventions or school support systems [11,12,54,55,56,57,58], lacking in-depth exploration of language interaction patterns within natural home environments and their micro-level connections to language abilities. Most studies stop at comparing group differences between CI preschool children and NH preschool children, failing to reveal how specific family language activities (such as activity type and frequency) concretely influence CI preschool children’s language development. Second, China’s unique family and cultural context renders this research gap particularly critical. The multigenerational childcare model, the linguistic environment where Mandarin and dialects coexist, and the cultural emphasis on early childhood education collectively shape complex family language input scenarios [59,60,61,62,63]. This implies that intervention programs developed based on Western monolingual nuclear family models may not be fully applicable. Therefore, to formulate localized, effective support strategies, the primary step is to systematically clarify the actual landscape of language activities within Chinese CI preschool children’s families and their relationship with language proficiency. Although studies indicate a positive correlation between language activity frequency and developmental levels [64], the specific activities undertaken, the methods employed, and the underlying mechanisms linking these activities to language abilities remain unclear within the Chinese context.

This study aimed to examine how the actual language activities in the home environment of hearing-impaired preschool children with CIs relate to their language abilities. Therefore, this study targets preschool children (aged 3–5) with CIs in China to: (1) first, through semi-structured interviews, identify the various language activities present in the home environment; (2) subsequently, analyze whether these activities exhibit distinct types; (3) based on this, quantitatively measure the frequency of occurrence for each type of language activity and other indicators of the home language environment; and (4) finally, validate the correlation between these language activity types and standardized language test results. By comparing these findings with research outcomes from NH preschool children, this study aimed to provide foundational data for developing culturally appropriate and effective support strategies.

## 2. Materials and Methods

### 2.1. Participants

This study included 118 CI preschool children and 135 NH preschool children as a control group aged 3–5 years. The CI group consisted of 68 males and 50 females, with a mean age of 4.50 years (standard deviation = 0.83 years). The average age at implantation for CI preschool children was 1.86 years (range: 0.5 to 3.75 years). The NH group consisted of 69 males and 66 females, with a mean age of 4.66 years (standard deviation = 0.83 years). The main characteristics of the participants are presented in Table 1.

The CI Group were systematically recruited from the rehabilitation centers(13 Institutes) for special education programs for hearing impaired children, in 12 locations including Huaihua City, Zhuzhou City and Changsha City in Hunan Province, Nanchang City and Ganzhou City in Jiangxi Province, Zhengzhou City in Henan Province, Kunming City in Yunnan Province, Baoding City in Hebei Province, Guangzhou City in Guangdong Province, Taizhou City in Zhejiang Province, Chongqing Municipality, and Beijing Municipality, the capital of China. Inclusion criteria: (1) Aged 3–5 years, (2) Bilateral cochlear implant users, (3) Given that auditory and language adaptation after cochlear implantation requires at least 6 to 12 months, and that children’s language perception abilities tend to stabilize 12 months after implantation [65], all preschool children with CIs had a minimum of one year of CI experience and had no other disabilities, (4) To ensure the absence of general developmental delays in the study groups, all preschool children were assessed using the Chinese version of The Ages & Stages Questionnaire, Third Edition (ASQ-3), a standardized developmental screening instrument designed for children aged 1–5 years and 11 months. The ASQ-3 evaluates child development across five distinct domains: communication, gross motor skills, fine motor skills, problem-solving, and personal-social development. Each domain consists of six items, with each item scored on a 10-point scale. This scoring system yielded a maximum possible score of 60 for each domain and a total overall score of 300. The questionnaires were filled out by parents, who provided responses based on structured observations of their children’s everyday behaviors. To ensure sample homogeneity, the study employed the following criteria for selection: For CI preschool children, those scoring significantly below the normative range in any domain of the Questionnaire (ASQ-3) except the communication domain (indicating potential coexisting developmental delays) were excluded. All 118 children met all criteria and were included. The Chinese version of the ASQ-3 demonstrated satisfactory psychometric properties in a normative Chinese sample, showing both strong reliability and validity. Internal consistency, measured using Cronbach’s alpha, was 0.80, indicating good item homogeneity [66]. Consequently, the psychometric findings warranted the application of the Chinese ASQ-3 to evaluate the developmental progress in this study. The NH Group was systematically recruited from a general education preschool in Jiangxi Province, China. Inclusion criteria: (1) Aged 3–5 years, (2) Among NH preschool children, hearing status was based on parental reports corroborated by school health records (specifically, annual physical examinations that included professional hearing screenings), with both sources indicating no known hearing concerns or other disabilities. (3) For NH preschool children, those scoring significantly below the normative range in any domain of the ASQ-3 were excluded. Accordingly, all 135 NH preschool children met the criteria and were fully included in the study. Additionally, to control for the critical confounding variable of family socioeconomic status and ensure that intergroup comparisons more clearly reveal the independent effects of hearing status (CI vs. NH) and home language environment, this study constructed the normal hearing control group (NH) using the principle of matching the socioeconomic level of families in the CI group. Specifically, from a broader pool of potential NH preschool children, we selected those whose SES indicators most closely matched the overall distribution of the CI group based on a comprehensive assessment of parental education level, occupation, and household income. This matching procedure aimed to minimize potential interference from SES on outcome variables, thereby enhancing the study’s internal validity. The details of the descriptive statistics and independent samples *t*-test results are presented in Table 2.

This study was approved by the approval of the Research Ethics Committee of the Faculty of Human Sciences of the University of Tsukuba. (Approval Number: Tsukuba 2024-176A) Written informed consent was obtained from the school principal, teachers, and parents of all participants.

### 2.2. Assessment of Language Ability

This study assessed participants’ language abilities using the Mandarin Clinical Evaluation of Language for Preschoolers’ Core Scale (MCELP-CS) [67]. The MCELP-CS was selected for the following key reasons: (1) the reliability and validity of this tool have been thoroughly verified in previous research [68], confirming that it meets psychometric standards (Cronbach’s α > 0.70, ICC > 0.75); (2) significant correlations between the MCELP-CS and PPVT-R showed a high convergent validity; (3) the development of this scale was grounded in the cultural and educational milieu of Chinese children’s early childhood setting; and (4) the scale also indicated good diagnostic accuracy in differentiating language disorders in children with autism, cerebral palsy, and hearing impairment.

The MCELP-CS consists of five subscales: vocabulary comprehension, sentence comprehension, vocabulary naming, sentence structure imitation, and story narration. The scale was used to measure the receptive and expressive language abilities of children aged 3–5 years and 11 months. The language assessment was administered individually to the rehabilitation center and in the kindergarten’s instruction room. Before administering the assessment, the examiner verbally explained the content in simple terms for approximately 5 min and conducted practice tasks. To ensure the quality of assessment and minimize the effects of fatigue, given the physical stamina and attention span of the participating children, the five subtests of the MCELP-CS were administered across three separate sessions following a fixed and identical sequence for all children: the first session included the Vocabulary Comprehension subtest (approximately 10–20 min); the second session consisted of the Sentence Comprehension and Vocabulary Naming subtests (approximately 15–20 min); and the third session comprised the Sentence Structure Imitation and Story Narration subtests (approximately 15–20 min). The total testing time ranged from approximately 40 to 60 min, distributed across different days.

### 2.3. Family Background and Language Activities Questionnaire in the Home Environment

To measure the frequency of language activities within the home and other related variables, this study developed a customized “Home language environment and activities survey questionnaire.” This questionnaire was designed based on a systematic review of domestic and international literature concerning the home language environment of NH preschool children and CI preschool children. To enhance its appropriateness within the context of Chinese culture and CI user families, researchers first conducted pre-interviews (approximately 15–20 min each) with 30 parents of preschool-aged CI preschool children and 30 parents of NH preschool children to explore the types of language activities actually occurring in their homes. Based on the literature review and interview findings [69], we developed an initial questionnaire comprising two main sections: background information and language activity frequency.

The content validity of the initial questionnaire was ensured through expert review. Based on feedback, redundant and ambiguous items were removed, ultimately retaining the 12 most representative language activity items. The assessment primarily covers the following four dimensions: (1) Frequency of language interaction: This evaluates communication between adults and children, as well as among children themselves. Examples include the extent to which primary caregivers communicate with your child, and whether your child actively asks questions or shares daily experiences with family members. (2) Parent–child shared activities: Evaluates literacy activities caregivers engage in with children, including shared reading and other literacy activities (e.g., How often does the primary caregiver engage in shared reading with the child? Beyond picture book reading, how often do you engage your child in other language-based learning activities at home? Play, outings, and joint household tasks. (3) Electronic media use: Investigates children’s use of electronic devices, such as televisions and mobile phones, including frequency and daily duration. A pilot test was conducted with six parents before the formal survey to ensure that the questions were clearly stated, unambiguous, and easy to understand.

To explore the latent structure of initial items and streamline the measurement tool, exploratory factor analysis (EFA) was conducted on 135 valid questionnaires randomly collected from parents of 3 to 5-year-old kindergarten children in China. The KMO test (0.83) and Bartlett’s sphericity test (χ^2^ = 801.43, df = 66, *p* < 0.001) indicated the data were suitable for factor analysis. To further simplify the model and enhance the scale’s validity, we optimized the items based on data analysis results. Principal component analysis (using maximum variance rotation) ultimately extracted two distinct factors based on eigenvalues, scree plots, and factor interpretability, retaining a total of nine items. During analysis, one item was removed due to low factor loadings and cross-loadings within its assigned factor. Additionally, to ensure model simplicity and robustness, the latent factor formed by the two items concerning children’s frequency and duration of electronic device use was discarded. This decision was made because a two-item factor is under-identified and statistically unstable, providing insufficient content coverage for a reliable measurement construct. These issues were confirmed during principal component and reliability analyses, leading to their removal. Factor 1 comprises four items, including parent–child reading, Chinese character writing, and storytelling, etc., named “Family literacy activities.” Factor 2 comprises five items, primarily covering activities such as daily conversations and question-and-answer sessions, and similar activities, named “Daily communication activities.” Together, these two factors explained 57.20% of the total variance. Subsequently, confirmatory factor analysis (CFA) was conducted using a separate sample of 210 valid questionnaires to test this two-factor model. The model fit was good: χ^2^(26) = 55.266, df = 26, χ^2^/df = 2.126, CFI = 0.948, TLI = 0.932, RMSEA = 0.073 (90% CI [0.046, 0.100]), SRMR = 0.056, and NNFI = 0.932. All standardized factor loadings were significant (range: 0.54–0.83). Regarding reliability and validity: The composite reliability (CR) for both factors was 0.79, with Cronbach’s α coefficients of 0.78 and 0.79, respectively, indicating good internal consistency. Although the average variance extracted (AVE) values for the two factors (0.437 and 0.488, respectively) were slightly below the ideal benchmark of 0.50, the measurement model’s convergent validity was deemed sufficient to support subsequent analyses. This conclusion was based on the satisfactory CR values, the high and significant factor loadings across all items, and the established discriminant validity (AVE square roots exceeding inter-factor correlations) [70,71,72].

Therefore, the final questionnaire comprises two sections. The first section gathered the participants’ background variables, including age, gender, presence of additional disabilities, age at cochlear implantation, hearing levels before and after implantation, status of CI use, family economic level, father’s and mother’s education level, and the number of books in the home(the number of books owned by the child and adults). To simplify the model and represent overall family resources, we combined relevant items into composite variables. First, father’s and mother’s educational levels were merged into a single “parental education level” variable (averaged; Cronbach’s α = 0.82, CITC > 0.69). Second, the number of children’s books and adult books at home was averaged into a composite “the number of books in the home” variable (Cronbach’s α = 0.70, CITC > 0.5). Both composites are based on 5-point Likert-scale items and were used in subsequent analyses. The second section assessed the variables related to the frequency of language activities. This consists of a two-dimensional scale (comprising the nine items mentioned above: family literacy activities, daily communication activities) scored on a 5-point Likert scale. The complete questionnaire is provided as Supplementary File S1 for review.

### 2.4. Data Analysis

This study aimed to investigate the association between family language activities and language abilities in children with CIs, using a control group of NH preschool children. All statistical analyses were performed using the IBM SPSS Statistics software (version 30.0). Descriptive statistics were conducted for all variables, with continuous variables expressed as mean ± standard deviation. Assuming a normal distribution, the Shapiro–Wilk test and Q-Q plots were used to assess the normality of the data. As the data for each age group and the overall data for both groups met the normality criteria, independent samples *t*-tests with a Bonferroni correction were conducted for group comparisons. Correlations between variables across both groups were assessed using Pearson’s rank correlation coefficient.

To further explore the relationship between language activities, other family variables, and language ability, stratified regression analyses were conducted separately for the CI and NH groups. Preliminary analysis revealed moderate to high correlations between parental education level, family income, and the number of books in the home (Pearson’s r range: 0.558–0.602), with variance inflation factors (VIFs) for variables in the regression model ranging from 1.5 to 2.1. To enhance model robustness, mitigate potential multicollinearity effects on parameter estimates, and reflect the theoretical consideration that these variables collectively indicate family socioeconomic status, we created a composite variable termed “Socioeconomic Resources” (SESR) measure from three ordinal indicators: highest parental education level, annual household income bracket, and the number of books in the home. To avoid the assumption of equal intervals between categories inherent in simple averaging, we employed principal component analysis (PCA). The data were suitable for dimension reduction, as indicated by a Kaiser-Meyer-Olkin measure of sampling adequacy of 0.71 and a significant Bartlett’s test of sphericity (*p* < 0.001). A single component with an eigenvalue greater than 1 (eigenvalue = [2.147]) was extracted, accounting for [71.55]% of the total variance. All items loaded strongly on this component (loadings: parental education level = 0.831, family income = 0.853, number of books = 0.853). The component scores from this first principal component were saved and used as our continuous SESR variable in all subsequent analyses. This approach provides a data-driven, weighted composite that reflects the shared variance among the indicators without imposing interval-scale assumptions on the original ordinal data. Subsequently, to more precisely assess the temporal effects of auditory intervention and in line with previous research indicating that “duration of CI use” is more directly associated with language development than “age at implantation” alone [73], we constructed the key variable “duration of CI use.” It is defined as: Duration of CI use = Chronological Age at Assessment—Age at cochlear implantation. This variable comprehensively reflects the cumulative period of effective auditory learning since implantation and serves as a more direct indicator of the “dose” effect of auditory rehabilitation. In the regression model, the contributions of family literacy activities and daily communication activities to language ability were examined for the CI group after controlling for age, SESR, and duration of CI use and for the NH group after controlling for age and SESR. Statistical significance for the regression models and predictor variables was set at *p* < 0.05. ΔR^2^ was used to assess the improvement in model explanatory power by adding new variables, and the contribution level of each predictor variable was represented by its standardized coefficient (*β*). The detailed analysis results are presented in the tables.

## 3. Results

### 3.1. Infant Development Test Screening (ASQ-3)

Descriptive statistics and independent samples *t*-test results on the Infant Development Test Screening stratified by age (3, 4, and 5 years) between 118 children with cochlear implants (CI group) and 135 children with normal hearing (NH group), encompassing multiple developmental domains including communication skills, motor skills, problem-solving, and personal-social skills (Table 2). All *p*-values underwent Bonferroni correction. The results revealed that in the communication domain, the NH group scored significantly higher than the CI group at all ages: at age 3 (CI group: M = 39.50, SD = 18.87; NH group: M = 56.78, SD = 3.72), *p* < 0.001; at age 4 (CI group: M = 40.38, SD = 15.45; NH group: M = 57.78, SD = 3.93), *p* < 0.001; at age 5 (CI group: M = 37.56, SD = 18.60; NH group: M = 58.33, SD = 2.82), *p* < 0.001. Regarding gross and fine motor skills, no significant differences were observed between groups across all age groups (all *p* > 0.05), indicating that motor development in CI preschool children is comparable to that of age-matched NH preschool children. In the problem-solving and personal-social development domains, although the NH group scored significantly higher than the CI group at all ages (all *p* < 0.05), both groups scored above 50 points and were classified as “developmentally normal” according to ASQ-3 criteria. Total score comparisons revealed that the NH group significantly outperformed the CI group across all age groups (*p* = 0.004 at age 3; *p* < 0.001 at ages 4 and 5). Where homogeneity of variance tests were significant, Welch’s *t*-test was employed (indicated in bold within tables).

### 3.2. Language Ability, Language Activity, and Other Family Variables

Descriptive statistics and the results of the independent samples *t*-tests comparing the CI group (n = 118) and the NH group (n = 135) across various variables stratified by age (3, 4, and 5 years) are presented in Table 3. When the assumption of homogeneity of variances was violated, Welch’s t-test was applied. To control for multiple comparisons across age strata, *p*-values were adjusted using the Bonferroni method. Language abilities and daily communication activities: This study compared the language abilities and daily communication performance of children with CI and NH peers across different age groups. In terms of language abilities, the NH group significantly outperformed the CI group at all three age levels with large effect sizes (3-year-olds: t (56.51) = 4.66, *p* < 0.001, d = 1.05; 4-year-olds: t (53.70) = 8.88, *p* < 0.001, d = 2.03; 5-year-olds: t (41.27) = 5.90, *p* < 0.001, d = 1.38). For daily communication activities, the NH group demonstrated significantly better performance at age 3 (t (83.00) = 3.59, *p* = 0.001, d = 0.78) and age 4 (t (82.00) = 2.47, *p* = 0.016, d = 0.54). However, by age 5, the difference between the two groups was no longer statistically significant after Bonferroni adjustment (t (61.77) = 1.58, *p* = 0.11, d = 0.36). Family socioeconomic resources and home literacy environment: In contrast, no significant differences were found between the CI and NH groups in any of the measures related to family socioeconomic resources or home literacy environment across the age strata. Specifically, independent samples t-tests revealed no statistically significant group differences in family economic level, parental education level, the number of books at home, or the composite SESR index at any age after Bonferroni adjustment (all *p* > 0.05). Similarly, no significant differences were found in family literacy activities at age 3 (t (83.00) = −0.33, *p* = 0.74, d = −0.07), age 4 (t (82.00) = −0.67, *p* = 0.51, d = −0.15), or age 5 (t (82.00) = −1.32, *p* = 0.19, d = −0.29). CI group characteristics: In the CI group, the mean age of cochlear implantation increased from 1.60 years (SD = 0.63) at age 3 to 2.03 years (SD = 0.92) at age 5. Correspondingly, the mean duration of CI use also increased across age groups, from 1.95 years (SD = 0.69) at age 3 to 3.42 years (SD = 0.94) at age 5.

Independent samples *t*-tests were used to compare differences between the CI group and the NH groups in terms of overall language ability, family background characteristics, and language activities. Detailed results are presented in Table 4.

Analysis revealed that, in terms of language ability, the NH group scored significantly higher than the CI group (t (184) = 9.83, *p* < 0.001, Cohen’s d = 1.28). The NH group also demonstrated a significant advantage in daily communication activities (t (218) = 4.45, *p* < 0.001, Cohen’s d = 0.57). By contrast, no significant differences were observed between the two groups across all measures of family socioeconomic resources. Specifically, both groups were comparable in terms of family economic status (t (251) = 1.56, *p* = 0.12), parental education level (t (251) = 0.64, *p* = 0.53), number of books at home (t (251) = −0.93, *p* = 0.35), and the composite socioeconomic resources index (SESR) (t (251) = 0.30, *p* = 0.77). Furthermore, no significant difference was found in the frequency of family literacy activities between the two groups (t (251) = −1.32, *p* = 0.19).

### 3.3. The Correlation Between Language Ability and Variables in the Home Environment

Table 5 presents the correlations between language abilities, and a set of demographic and home environment variables for the sample of the CI group (n = 118).

CI preschool children’s language abilities demonstrated statistically significant correlations with all investigated variables. Notably, language abilities showed a strong positive correlation with the duration of CI use (r = 0.509, *p* < 0.01) and daily communication activities (r = 0.507, *p* < 0.01). Moderate positive correlations were observed between language abilities and the composite index of family socioeconomic resources (SESR) (r = 0.380, *p* < 0.01), the number of books in the home (r = 0.379, *p* < 0.01), family literacy activities (r = 0.353, *p* < 0.01), parental education level (r = 0.308, *p* < 0.01), and family economic level (r = 0.300, *p* < 0.01). A significant positive correlation was also found between language abilities and the children’s age (r = 0.285, *p* < 0.01). In contrast, a significant negative correlation was observed between language abilities and the age of cochlear implantation (r = −0.358, *p* < 0.01), indicating that earlier implantation was associated with higher language scores. Furthermore, the correlation matrix revealed several other significant associations among the predictor variables. The duration of CI use was strongly correlated with the child’s age (r = 0.593, *p* < 0.01) and, as expected, strongly negatively correlated with the age of implantation (r = −0.599, *p* < 0.01). The composite SESR index was very strongly correlated with its components: parental education level (r = 0.912, *p* < 0.01), the number of books in the home (r = 0.866, *p* < 0.01), and family economic level (r = 0.814, *p* < 0.01). Daily communication activities were significantly correlated with family literacy activities (r = 0.563, *p* < 0.01) and all SESR components (rs ranging from 0.302 to 0.401, *p* < 0.01).

The results of the correlation analysis between language ability and family environment variables for NH preschool children (n = 135) are presented in Table 6.

Compared to CI preschool children, NH preschool children exhibited distinct patterns of correlations between language abilities. Specifically, language ability showed a highly significant positive correlation with child age (r = 0.751, *p* < 0.01). Regarding family socioeconomic resources (SESR), language ability was significantly positively correlated with the composite SESR index (r = 0.275, *p* < 0.01). Specifically examining the components of SESR, language ability showed significant positive correlations with the number of books in the home (r = 0.255, *p* < 0.01) and parental education level (r = 0.240, *p* < 0.01). However, the correlation with family economic level did not reach statistical significance (r = 0.137, *p* > 0.05). In terms of home language environment, language ability showed a significant positive correlation with family literacy activities (r = 0.226, *p* < 0.05) but no significant association with daily communication activities (r = 0.085, *p* > 0.05). Furthermore, the correlation matrix revealed other significant associations among the variables. The composite SESR index was very strongly correlated with its components: family economic level (r = 0.784, *p* < 0.01), parental education level (r = 0.863, *p* < 0.01), and the number of books in the home (r = 0.846, *p* < 0.01). Daily communication activities were significantly correlated with family literacy activities (r = 0.476, *p* < 0.01) and all SESR components (ranging from 0.230 to 0.393, *p* < 0.01).

### 3.4. The Impact of All Variables in the Home Environment Relates to Language Ability

A hierarchical regression analysis was conducted to predict language abilities in CI preschool children (n = 118) (Table 7).

Model 1 included Age and the composite socioeconomic resources index (SESR). The model was statistically significant, F = 17.334, *p* < 0.001, and explained 23.2% of the variance in language abilities (Adjusted R^2^ = 0.218). Both Age (β = 0.30, t = 3.61, *p* < 0.001) and SESR (β = 0.39, t = 4.74, *p* < 0.001) were significant positive predictors. Model 2 added Duration of CI use. This model explained 32.7% of the variance (Adjusted R^2^ = 0.309), representing a significant increase in explanatory power (ΔR^2^ = 0.095, F = 16.057, *p* < 0.001). In this model, the Duration of CI use emerged as a strong positive predictor (β = 0.41, t = 4.01, *p* < 0.001). SESR remained a significant positive predictor (β = 0.28, t = 3.37, *p* = 0.001). Notably, Age, which was significant in Model 1, became non-significant after controlling for Duration of CI use (β = 0.05, t = 0.52, *p* = 0.605). Model 3 further added Family literacy activities. This model explained 35.4% of the variance (Adjusted R^2^ = 0.331), with a significant increase (ΔR^2^ = 0.028, F = 4.843, *p* = 0.03). In this model, Duration of CI use (β = 0.39, t = 3.85, *p* < 0.001) and SESR (β = 0.21, t = 2.50, *p* = 0.014) remained significant positive predictors. Family literacy activities also emerged as a significant positive predictor (β = 0.18, t = 2.20, *p* = 0.030). Age remained non-significant (β = 0.04, t = 0.45, *p* = 0.655). Model 4 finally added Daily communication activities. The final model explained 41.4% of the variance (Adjusted R^2^ = 0.387), with a significant increase (ΔR^2^ = 0.059, F = 11.332, *p* = 0.001). In this final model, Duration of CI use (β = 0.35, t = 3.65, *p* < 0.001) and Daily communication activities (β = 0.31, t = 3.37, *p* = 0.001) were strong and significant positive predictors. Notably, both SESR (β = 0.15, t = 1.78, *p* = 0.078) and Family literacy activities (β = 0.04, t = 0.45, *p* = 0.655), which were significant in previous models, became non-significant after controlling for Daily communication activities. Age remained non-significant (β = 0.02, t = 0.20, *p* = 0.842).

A hierarchical regression analysis was performed to predict language abilities in normal-hearing (NH) preschool children (n = 135) (Table 8).

Model 1 included Age and the composite socioeconomic resources index (SESR). The model was statistically significant, F = 98.288, *p* < 0.001, and explained 59.8% of the variance in language abilities (Adjusted R^2^ = 0.592). Age was an exceptionally strong positive predictor (β = 0.73, t = 13.15, *p* < 0.001), and SESR was also a significant positive predictor (β = 0.19, t = 3.34, *p* = 0.001). Model 2 added Daily communication activities. This model explained 59.9% of the variance (Adjusted R^2^ = 0.590), representing a non-significant increase in explanatory power (ΔR^2^ = 0.001, F = 0.398, *p* = 0.529). In this model, Age (β = 0.73, t = 13.13, *p* < 0.001) and SESR (β = 0.17, t = 2.88, *p* = 0.005) remained significant predictors. Daily communication activities was not a significant predictor (β = 0.04, t = 0.63, *p* = 0.529). Model 3 further added Family literacy activities. The final model explained 62.2% of the variance (Adjusted R^2^ = 0.610), with a significant increase (ΔR^2^ = 0.022, F = 7.597, *p* = 0.007). In this final model, Age remained the strongest and most robust predictor (β = 0.73, t = 13.41, *p* < 0.001). Family literacy activities emerged as a significant positive predictor (β = 0.17, t = 2.76, *p* = 0.007). SESR retained its significance, though its predictive weight slightly decreased (β = 0.15, t = 2.47, *p* = 0.015). Notably, Daily communication activities remained non-significant (β = −0.03, t = −0.54, *p* = 0.589).

Summary of Key Contrast

The final regression models revealed a divergent pattern of significant predictors between the two groups. For children with CIs, daily communication activities were a significant predictor, whereas for NH preschool children, family literacy activities were significant.

## 4. Discussion

This comparative study examined the relationship between home language activities, related variables, and language ability in CI preschool children and their NH peers. Core findings indicate that while supportive home environments are crucial for both groups, the importance of daily communication and family literacy activities follows distinctly opposite patterns for CI and NH preschool children. This suggests the need for tailored educational approaches for different groups. It is important to emphasize, however, that this study primarily reveals correlations between variables, the underlying causal direction or mechanisms require further investigation.

Summary and key findings: Differentiated patterns of association

First, consistent with expectations, the NH group significantly outperformed the CI recipients on standardized language assessments across all age groups. This result suggests that continuous support is necessary for preschool children with CIs to catch up with their NH peers during language development [74]. Furthermore, Correlation analysis revealed a negative correlation between language ability and age at implantation, and a strong positive correlation with CI duration of use. These findings reaffirm the principle of auditory neural plasticity and the critical importance of early intervention, as infants implanted at younger ages demonstrated superior language skills [19]. However, hierarchical regression analysis uncovered a more nuanced mechanism: after CI duration of use was incorporated into the model, the predictive effect of age was no longer significant. This strongly suggests that for CI preschool children, cumulative, high-quality auditory experience—characterized by both duration of use and daily interaction—rather than mere physiological maturation or a singular early implantation time point, serves as the core engine driving their language development. This aligns with the understanding that as chronological age increases, it is the accumulated auditory experience gained over time that primarily promotes neural plasticity and supports ongoing language acquisition [17]. Thus, while early implantation initiates the process, it is the consistent and rich auditory input within the critical period that plays the decisive role in optimizing outcomes, reinforcing the comprehensive principle of “early detection, early intervention, early rehabilitation”. This study did not find significant differences in family socioeconomic resources between the two groups of children, providing a comparable basis for subsequent analyses. However, regression analysis revealed that the same resource background might influence language development differently for the two groups. Among NH preschool children, socioeconomic resources demonstrated a direct and stable enhancing effect. In contrast, among CI preschool children, the explanatory power of the final model primarily derived from auditory experience (duration of CI use) and interaction quality (daily communication activities). The independent contribution of socioeconomic resources became non-significant after controlling daily communication activities (β = 0.15, *p* = 0.078). This suggests that for CI preschool children, family socioeconomic resources may primarily influence language ability by facilitating high-quality daily language interactions as a mediating pathway, rather than exerting a direct effect. This difference further highlights the heightened reliance of CI preschool children’s language development on specific experiential factors (auditory input and interaction), suggesting that their developmental trajectory may be more “experience-sensitive” rather than “direct resource-transforming” compared to that of NH preschool children.

However, a subtle pattern emerged in the developmental trajectories of specific language activity: while NH preschool children showed advantages in everyday communicative activities at ages 3 and 4, this difference had disappeared by age 5. This suggests that with technological intervention and rehabilitation, children with CI can achieve functional catch-up in foundational interactive skills finding consistent with several long-term follow-up studies [7,19]. In other words, by age 5, both groups of children performed comparably in daily communication. With scientific intervention, preschool children with CI can catch up. However, gaps persisted in more complex, cognitively demanding language abilities. These include skills such as narrative production (telling a coherent story), understanding and using complex syntax, advanced vocabulary for abstract concepts, and inferential comprehension. This pattern may indicate that after establishing a solid functional “foundation” in basic communication, children with CIs require targeted, higher-level linguistic stimulation to “build” the more elaborate cognitive–linguistic structures necessary for subsequent learning and social development.

However, the most striking findings emerged from the correlation and regression analyses, which revealed distinctly different predictive association models for the two groups of children. The regression model constructed in this study indicates that the overall explained variance for the CI group was 39.5%, while that for the NH group reached 60.7%. Among NH preschool children, age was the primary predictor of developmental outcomes. This dominant role of age in the NH group warrants further discussion. The high explanatory power of age likely captures the robust, cumulative effect of typical neuro-maturation and consistent, daily language exposure that all NH preschool children experience. In such a context, the additional, unique contribution of specific family language activities might be statistically “masked” or appear smaller, as its influence is partly embedded within the general developmental trajectory indexed by age. This is not to say family environment is unimportant for NH preschool children, rather, its effect may be more homogenized and intertwined with maturational gains. In the hierarchical regression, after controlling for age, home literacy activities still explained a significant portion of additional variance, confirming it as a significant predictor. It is noteworthy that, even when age accounted for the majority of the variance, family socioeconomic resources (SESR) consistently emerged as a significant independent predictor (*p* < 0.05 in all models). This suggests that while language development in NH preschool children is highly dependent on age-related neural maturation and general language exposure, the economic, educational, and cultural capital available to the family can provide additional gains beyond the baseline developmental trajectory. This finding aligns with multiple theories in developmental science, which posit that advantageous resources can promote children’s cognitive and linguistic development by offering richer learning materials, higher-quality educational opportunities, and more cognitively stimulating family interaction environments [75]. Conversely, for CI preschool children, the developmental pathway is more variable and less assured by chronological age alone. Factors like the timing of auditory access (age at implantation), CI duration of use, the intensity and quality of post-implantation rehabilitation, and deliberate family engagement become critical, active drivers of development, explaining variance beyond what simple age can predict. Therefore, the observed differences in the models reveal a fundamental distinction: the development of NH preschool children primarily follows an age-related maturational pattern, whereas the developmental trajectory of CI preschool children more clearly demonstrates that their neural plasticity is highly dependent on the accumulation of effective auditory experience—objectively measured by CI duration of use—as well as the quality of that experience, such as the level of daily communicative interaction.

For the CI group, stratified regression analysis revealed that daily communication activities exhibited the most robust and unique positive association with language ability, accounting for significant additional variance even after controlling for age, age at CI implantation, and socioeconomic resources. Conversely, when daily communication activities were included, the unique association between family literacy activities and language ability in children with CIs ceased to be significant. This may indicate that for this group, the language interaction elements encompassed by daily communication are more fundamental and pervasive. In NH preschool children, however, home literacy activities demonstrated a clearer independent association.

Simply put, for CI preschool children, daily communication activities become stronger predictors because they occur during the critical foundational period of auditory and language development. These interactions provide dense, contextualized language input that requires clear articulation—essential for consolidating auditory perception and building basic vocabulary. Once this fundamental need is met, the additional contribution of more structured family literacy activities may be “masked” or relatively diminished. In other words, for CI preschool children, engaging in daily chats and playful conversations with them boosts language development more effectively than dedicated reading and letter recognition activities. Thus, the repetitive, everyday exchanges that occur spontaneously throughout daily life (like describing dishes during meals, explaining steps while dressing, or responding to their sounds during play) become the most potent and intensive “hearing rehabilitation training.” During this critical stage, the quality and quantity of daily interactions directly foster foundational language skills more effectively than specialized literacy activities. Conversely, NH preschool children effortlessly absorb vast amounts of language from their environment. Thus, purposeful, cognitively challenging family literacy activities beyond routine communication, such as shared reading and story narration, have become key factors in further stimulating their language complexity and metalinguistic awareness. In other words, for NH preschool children, engaging in conscious literacy activities, such as parent–child reading and storytelling, provides more pronounced benefits for language development, while the role of everyday chatting is less significant.

This pattern may be interpreted through the lens of “developmental demand prioritization.” The NH group may possess earlier maturity in processing everyday language input, making the organized, decontextualized language stimulation provided by family literacy activities more closely linked to their higher-level language skill development. Conversely, the CI group faces initial challenges in perceiving and processing speech signals. The dense, contextual, and highly interactive language exposure provided in daily communication may align more closely with their core need to build foundational language representations, thus showing a stronger correlation. Simultaneously, we observe a moderate correlation between these two activities, suggesting that they often co-occur in the home environment and may share common components that promote language development.

This study found that the predictive patterns of family activities on language abilities differ across groups: among NH preschool children, structured and content-rich “language-learning activities” (such as shared reading) are stronger predictors; while among children with cochlear implants, the frequency of “daily communication” demonstrates stronger predictive power. This difference may stem from two interrelated dimensions: First, at the behavioral level, it may reflect variations in the language development pathways of different child populations. NH preschool children, after receiving sufficient foundational language input, may rely more on guided, cognitively engaging interactions for further language development; whereas children with cochlear implants are in a critical foundational period for auditory and language development, where high-frequency, sustained daily auditory and linguistic input is particularly important for establishing their language systems. Second, at the measurement and psychological level, this difference may also partly reflect variations in how families perceive and report similar activities. For parents of children with cochlear implants, “daily communication” is often closely linked to rehabilitation goals. When reporting its frequency, they may subconsciously assess the attentiveness and quality of linguistic input, making this measurement effectively encompass a construct of “high-engagement family intervention.” In contrast, parents of NH preschool children may report it more purely as natural social frequency. Therefore, the “daily communication” factor in this study may capture constructs that differ in both behavioral intensity and psychological significance across the two groups.

Prudent interpretation of correlation and potential causality

We interpret these relationships with caution. First, significant correlations alone cannot establish causation. One plausible explanation is that engaging in abundant family language activities promotes children’s language development. However, a reverse causal pathway is equally plausible: children with stronger language abilities may be more likely to initiate or engage in higher-quality, more frequent family interactions. The cross-sectional data and correlation/regression analyses employed in this study cannot rigorously distinguish between these two directions. Therefore, we present our findings as indicating a strong association or predictive relationship between language activities and language proficiency.

Nevertheless, these associative patterns hold significant indicative value. For instance, in NH preschool children, the auditory-linguistic foundation is established unconsciously, making the language encountered in daily communication an omnipresent and sufficiently robust foundation. The primary engine driving advanced language growth may then shift toward more specialized, cognitively demanding interactions—such as shared reading—which introduce complex vocabulary, narrative structures, and decontextualized language. This aligns with perspectives emphasizing the quality of home language input [27] and the role of cognitive challenges in typical development [28].

For CI preschool children, the situation differs. Despite technological intervention, auditory signals remain degraded compared to normal hearing, and the early post-implantation period coincides with a critical window for oral language neural and perceptual development [17,18]. Within this context, frequent, high-quality, and responsive daily interactions may serve as a crucial intensive “training ground.” It provides the substantial, repetitive, and contextualized exposure needed to consolidate phonological representations, foundational vocabulary, and syntactic patterns. Before this fundamental auditory-linguistic foundation is firmly established, the additional benefits from more structured literacy activities may be minimal or indistinguishable from the general communicative environment. This finding supports and refines intervention models that prioritize encouraging family engagement in responsive interactions and auditory enrichment. Second, this study found no significant differences in family socioeconomic resources between the two groups of children. This key control variable indicates that the observed differences in language development trajectories are more likely rooted in fundamental variations in auditory perception pathways rather than inequalities in family background. This finding strengthens the internal validity of the core discovery, reinforcing the inference that the observed differences in association patterns stem more from auditory experience itself than from resource inequality.

## 5. Research Implications and Limitations

At the same time, we must clearly acknowledge certain limitations when interpreting these findings. This study reveals correlations rather than definitive causal relationships. These discoveries provide an important empirical foundation and specific hypotheses for subsequent longitudinal studies and intervention experiments aimed at validating causal mechanisms. First, cross-sectional designs cannot infer causality. Longitudinal studies are needed to track how the effects of different family activities within the same child change over time. Second, key variables, including language activities, were measured through parent reports, which may be subject to social desirability bias. Future research would benefit from incorporating objective measures, such as LENA recordings, to more accurately capture the home language environment. Third, although we controlled several important factors, other unmeasured variables—such as the quality of rehabilitation services, and specific characteristics of cochlear implant devices and their tuning—may influence the results. Future Research Directions: “Future studies could adopt longitudinal designs to track the long-term effects of these activities; incorporate objective measurement tools (e.g., language environment recordings) to reduce reporting bias; and further explore the specific cognitive mechanisms through which these activities influence language abilities.”

Although the direction of causality remains to be determined, the robust association provides a valuable focus for family support and early intervention. It suggests that support strategies may need to be tailored to the child’s primary mode of communication. This contrasting predictive model offers clear and distinct implications for family-centered practice and guidance. For families of children with cochlear implants, particularly in the early post-implantation period, clinicians and educators should strongly emphasize strategies to increase both the quantity and quality of daily interactive communication. This entails encouraging abundant, clear, and child-interest-driven conversations across all daily contexts—such as mealtimes, hygiene routines, and play—transforming life into a continuous, enjoyable auditory-linguistic learning environment. For families of NH preschool children and early childhood educators: While ensuring abundant daily conversation, focus should be placed on promoting and guiding high-quality shared reading and discussion—deep literacy interactions that effectively catalyze language development toward higher levels.

One limitation of this study lies in the method used to confirm hearing status among children in the normal-hearing (NH) control group. Although we employed a dual-criterion approach combining “parent reports” with “professional hearing screening results from annual school health examinations” to verify their hearing status—a method more reliable than relying solely on parent reports—this approach remains fundamentally a screening tool rather than a comprehensive audiological diagnosis. Previous studies have indicated that parental assessments of their children’s hearing issues may be biased, often underestimating actual hearing loss [76]. Although school-based annual screening provides a professional and objective supplement, standardized pure-tone audiometry screening conducted in a controlled acoustic environment was not implemented in this study. Therefore, the possibility of unidentified mild or unilateral hearing loss within the control group cannot be entirely ruled out. This limitation suggests that future studies, when feasible, should conduct standardized audiological assessments for all participants (including the control group). This would more rigorously ensure the accuracy of intergroup classification and enhance the robustness of research conclusions. Additionally, the “the number of books at home” indicator used in this study is a static resource proxy variable that cannot fully capture the dynamic behaviors and quality of parent–child reading at home (for example, it does not reflect the use of public libraries). Nevertheless, this indicator is widely used in child development research as an effective and stable proxy for measuring family cultural capital and the learning environment, it also has a robust correlation with children’s language, cognitive, and academic development outcomes. [77,78]. In this study design, it primarily serves as a control variable to partially exclude the potential influence of family cultural resource background in the analysis. Future research should employ more direct behavioral measurement methods to deepen understanding of this environmental factor. Additionally, this study has certain limitations regarding its measurement model. Although electronic media use was considered a potentially relevant environmental variable, it was measured with only two items, which is insufficient to meet the basic identification requirements for a latent variable in structural equation modeling (a minimum of three items is necessary). Consequently, it could not be included as a formal latent variable in the analysis. This may have resulted in the model not fully capturing the potential influence of media use environment on the core constructs. Future research could develop or employ multi-item scales with stronger content validity to measure this variable more adequately and stably, and to further examine its role within the research framework. Furthermore, this study primarily relies on parental reports of the frequency of behaviors and does not differentiate between the quality and structural characteristics of the interactions. Additionally, the same questionnaire items may hold different ecological validity and interpretations across different groups (e.g., CI and NH families), limiting the construct equivalence of the measurement tool. Future research could employ observational methods or experience sampling methods to simultaneously capture the frequency, quality, and structure of family interactions, thereby providing more precise interpretations.

## 6. Conclusions

In summary, this study found that the language abilities of CI preschool children and NH preschool children exhibit distinct patterns of strong association with different types of family language activities. Overall language abilities in CI preschool children remain behind those of NH preschool children, with earlier implantation age correlating with stronger language skills—once again underscoring the critical importance of early intervention. Although CI preschool children achieve functional catch-up in everyday communication by age 5, gaps persist in complex language skills. This study further revealed that this functional catch-up is closely associated with the cumulative duration of CI use. With increased wearing time, children have more opportunities to practice and process auditory and linguistic information in naturalistic settings, thereby bridging gaps in foundational interactive skills. These findings provide empirical support for the concept of “experience-driven catch-up. For NH preschool children, language ability is primarily driven by age-related maturation while consistently benefiting from the systematic advantages conferred by family socioeconomic resources. Family literacy activities, in turn, serve as a key source of cognitive stimulation within this developmental process. Conversely, the developmental trajectory of the CI group suggests that their language abilities are primarily driven by cumulative auditory experience (CI duration of use) and the quality of that experience (frequent daily communicative activities). Even after controlling for family socioeconomic background, both factors continued to demonstrate strong independent predictive power. This indicates that their development exhibits a high degree of sensitivity and dependence on specific, modifiable experiential factors.

Examining the impact of language activities reveals that, in the CI group, family literacy activities (e.g., shared reading) were associated with language skills, but daily communication activities (e.g., conversational interactions) showed a more significant predictive effect on language abilities, maintaining this advantage even after controlling key demographic variables. Conversely, for NH preschool children, family literacy activities demonstrated a clearer independent association with language development. This study offers clearer insights for home-based rehabilitation practices among CI preschool children, indicating that interventions should prioritize high-quality daily communication (e.g., open-ended questioning, extended dialog, contextualized interactions) rather than over-reliance on structured literacy activities. It also indicates that intervention models designed for NH preschool children cannot be simply applied.

## Figures and Tables

**Table 1:** Participants’ information.

		CI Preschool Children (n = 118)	NH Preschool Children (n = 135)
Age	3	40	45
	4	39	45
	5	39	45
Gender	Boys	68 (57.62%)	69 (51.11%)
	Girls	50 (42.38%)	66 (48.89%)
Average Age		4.50 (0.83)	4.66 (0.83)
CI Age of Implantation		1.86 (0.79)	-
CI Status (Bilateral)		118 (100.00%)	

Note: CI: cochlear implant; NH: normal-hearing. Age range for CI placement (minimum age 0.5 years and maximum age 3.75 years).

**Table 2:** Descriptive statistics and independent samples *t*-test results for group differences on the Infant Development Test Screening stratified by age.

		CI Group (n = 118)		NH Group (n = 135)							
	Age	Mean (SD)	N	Mean (SD)	N	Levene’s Test	*t*	df	*p*-Value	Adjusted *p*-Value	Effect Size
Communications	3	39.50 (18.87)	40	56.78 (3.72)	45	**71.67**	5.69	41.69	0.000 **	0.000 **	1.31
	4	40.38 (15.45)	39	57.78 (3.93)	45	**45.48**	6.84	42.26	0.000 **	0.000 **	1.60
	5	37.56 (18.60)	39	58.33 (2.82)	45	**101.37**	6.91	39.52	0.000 **	0.000 **	1.62
Gross motor skills	3	56.13 (4.46)	40	56.78 (4.01)	45	0.50	0.71	83.00	0.48	0.48	0.15
	4	56.54 (4.32)	39	57.56 (2.52)	45	**4.50**	1.24	65.60	**0.22**	0.21	0.28
	5	56.54 (5.02)	39	57.67 (2.52)	45	**14.10**	1.27	54.21	**0.21**	0.19	0.29
Fine motor skills	3	53.75 (5.75)	40	55.11 (4.33)	45	**8.14**	1.22	72.03	**0.23**	0.22	0.27
	4	55.13 (5.06)	39	55.89 (3.89)	45	**6.88**	0.76	70.77	**0.45**	0.44	0.17
	5	56.28 (4.96)	39	57.44 (3.47)	45	3.60	1.26	82.00	0.21	0.21	0.28
Problem-solving development	3	51.53 (9.70)	40	55.00 (3.99)	45	**75.90**	2.05	50.59	0.045 *	0.035 *	0.47
	4	53.08 (7.58)	39	56.22 (3.87)	45	**37.56**	2.34	54.69	0.023 *	0.017 *	0.54
	5	52.95 (10.37)	39	57.44 (3.30)	45	**49.97**	2.60	44.69	0.013 *	0.007 **	0.60
Personal-social development	3	51.00 (10.99)	40	55.44 (3.96)	45	**69.58**	2.42	47.97	0.019 *	0.013 *	0.55
	4	53.08 (8.40)	39	57.00 (3.75)	45	**34.51**	2.69	50.98	0.010 **	0.006 **	0.62
	5	53.59 (8.73)	39	58.11 (2.67)	45	**55.15**	3.11	44.17	0.003 **	0.001 **	0.72
	3	252.00 (44.34)	40	279.11 (14.19)	45	**57.83**	3.70	46.09	0.001 **	0.000 **	0.84
Total scores	4	258.21 (36.28)	39	284.44 (12.80)	45	**40.64**	4.29	46.18	0.000 **	0.000 **	0.99
	5	252.31 (42.67)	39	289.00 (11.41)	45	**66.45**	5.21	42.72	0.000 **	0.000 **	1.21

Note: * *p* < 0.05. ** *p* < 0.01. CI: cochlear implant; NH: normal-hearing. When the assumption of homogeneity of variances was violated (as indicated by a significant Levene‘s test), Welch’s *t*-test was used instead of the standard Student’s *t*-test. In the results table, the values for these cases are presented in bold type. *p*-values were adjusted for multiple comparisons using the Bonferroni method.

**Table 3:** Descriptive statistics and independent samples *t*-test results for group differences on variables stratified by age.

		CI Group (n = 118)		NH Group (n = 135)							
	Age	Mean (SD)	N	Mean (SD)	N	Levene’s Test	*t*	df	*p*-Value	Adjusted *p*-Value	Effect Size
Language abilities	3	89.71 (49.86)	40	130.50 (25.48)	45	**26.37**	4.66	56.51	0.000 **	0.000 **	1.05
	4	105.51 (42.70)	39	172.30 (21.10)	45	**18.73**	8.88	53.70	0.000 **	0.000 **	2.03
	5	127.83 (64.24)	39	191.08 (14.46)	45	**94.94**	5.90	41.27	0.000 **	0.000 **	1.38
CI age of implantation	3	1.60 (0.63)	40	−	−	−	−	−	−	−	−
	4	1.95 (0.77)	39								
	5	2.03 (0.92)	39								
Duration of CI use	3	1.95 (0.69)	40	−	−	−	−	−	−	−	−
	4	2.56 (0.82)	39								
	5	3.42 (0.94)	39								
Family economic level	3	2.38 (0.98)	40	2.71 (0.97)	45	2.53	1.59	83.00	0.12	0.12	0.35
	4	2.51 (1.25)	39	2.56 (1.04)	45	1.76	0.17	82.00	0.87	0.87	0.04
	5	2.54 (1.35)	39	2.87 (1.14)	45	1.50	1.03	82.00	0.31	0.31	0.23
Parental education level	3	5.27 (2.15)	40	5.40 (2.06)	45	0.12	0.27	83.00	0.79	0.79	0.06
	4	5.31 (2.38)	39	4.98 (2.10)	45	1.03	−0.68	82.00	0.50	0.50	−0.15
	5	4.90 (2.53)	39	5.78 (2.22)	45	1.37	1.43	82.00	0.16	0.16	0.31
The number of books in the home	3	5.10 (2.02)	40	4.38 (1.90)	45	0.01	−1.70	83.00	0.09	0.09	−0.37
	4	4.90 (1.80)	39	4.89 (1.66)	45	0.82	−0.02	82.00	0.98	0.98	−0.01
	5	5.00 (2.32)	39	5.04 (2.07)	45	0.78	0.09	82.00	0.93	0.93	0.02
Composite Index (SESR)	3	12.75 (4.53)	40	12.49 (4.16)	45	0.50	−0.28	83.00	0.78	0.78	−0.06
	4	12.72 (4.58)	39	12.42 (3.96)	45	0.83	−0.32	82.00	0.75	0.75	−0.07
	5	12.44 (5.60)	39	13.51 (4.79)	45	2.31	0.95	82.00	0.35	0.35	0.21
Daily communication activities	3	17.10 (3.80)	40	19.82 (3.19)	45	0.89	3.59	83.00	0.001 **	0.001 **	0.78
	4	17.95 (3.50)	39	19.69 (2.97)	45	0.97	2.47	82.00	0.016 *	0.016 *	0.54
	5	18.74 (3.60)	39	19.69 (2.35)	45	**8.85**	1.58	61.77	**0.12**	0.11	0.36
Family literacy activities	3	12.40 (3.11)	40	12.18 (3.05)	45	0.02	−0.33	83.00	0.74	0.74	−0.07
	4	13.21 (2.93)	39	12.76 (3.21)	45	2.26	−0.67	82.00	0.51	0.51	−0.15
	5	13.15 (3.35)	39	12.36 (2.63)	45	1.48	−1.32	82.00	0.19	0.19	−0.29

Note: * *p* < 0.05. ** *p* < 0.01. CI: cochlear implant; NH: normal-hearing. When the assumption of homogeneity of variances was violated (as indicated by a significant Levene‘s test), Welch’s *t*-test was used instead of the standard Student’s *t*-test. In the results table, the values for these cases are presented in bold type. *p*-values were adjusted for multiple comparisons using the Bonferroni method. SESR = Composite index of family socioeconomic resources. This composite variable combines measures of family economic level, parental education, and the number of books in the home.

**Table 4:** Descriptive statistics and independent samples *t*-test for group differences on variables.

	CI Group Mean (SD)	NH Group Mean (SD)	Levene’s Test	*t*	df	*p*-Value	Adjusted *p*-Value	Effect Size
Language abilities	107.53 (54.85)	164.21(32.41)	**32.53**	9.83	184.35	<0.001 **	0.000 **	1.28
CI age of implantation	1.86 (0.79)	−	−	−	−	−		−
Duration of CI use	2.64 (1.02)	−	−	−	−	−		−
Family economic level	2.47 (1.20)	2.70 (1.06)	1.70	1.56	251.00	0.12	0.12	0.20
Parental education level	5.16 (2.34)	5.34 (2.15)	1.36	0.64	251.00	0.53	0.53	0.08
The number of books in the home	5.00 (2.05)	4.77 (1.89)	0.32	−0.93	251.00	0.35	0.35	0.12
Composite index (SESR)	12.64 (4.88)	12.81 (4.32)	2.81	0.30	251.00	0.77	0.77	0.04
Daily communication activities	17.92 (3.67)	19.77 (2.81)	**9.89**	4.45	217.77	<0.001 **	0.000 **	0.57
Family literacy activities	12.92 (3.13)	12.41 (2.98)	0.02	−1.32	251.00	0.19	0.19	0.17

Note: ** *p* < 0.01. CI: cochlear implant; NH: normal-hearing. When the assumption of homogeneity of variances was violated (as indicated by a significant Levene‘s test), Welch’s *t*-test was used instead of the standard Student’s *t*-test. In the results table, the values for these cases are presented in bold type. *p*-values were adjusted for multiple comparisons using the Bonferroni method. SESR = Composite index of family socioeconomic resources. This composite variable combines measures of family economic level, parental education, and the number of books in the home.

**Table 5:** The correlation between language abilities and variables in the home environment for preschool children with cochlear implants (n = 118).

	1	2	3	4	5	6	7	8	9	10
1. Language abilities	1									
2. Age	0.285 **	1								
3. CI age of implantation	−0.358 **	0.224 *	1							
4. Duration of CI use	0.509 **	0.593 **	−0.599 **	1						
5. Family economic level	0.300 **	0.056	−0.295 **	0.320 **	1					
6. Parental education level	0.308 **	−0.066	−0.290 **	0.162	0.677 **	1				
7. The number of books in the home	0.379 **	−0.02	−0.295 **	0.247 **	0.583 **	0.635 **	1			
8. Composite index (SESR)	0.380 **	−0.026	−0.336 **	0.260 **	0.814 **	0.912 **	0.866 **	1		
9. Daily communication activities	0.507 **	0.184 *	−0.204 *	0.313 **	0.302 **	0.342 **	0.389 **	0.401 **	1	
10. Family literacy activities	0.353 **	0.099	−0.172	0.226 *	0.15	0.319 **	0.435 **	0.372 **	0.563 **	1

Note: * *p* < 0.05. ** *p* < 0.01. CI: cochlear implant. SESR = Composite index of family socioeconomic resources. This composite variable combines measures of family economic level, parental education, and the number of books in the home.

**Table 6:** The correlation between language abilities and variables in the home environment for normal-hearing children (n = 135).

	1	2	3	4	5	6	7	8
1. Language abilities	1							
2. Age	0.751 **	1						
3. Family economic level	0.156	0.061	1					
4. Parental education level	0.233 **	0.072	0.559 **	1				
5. The number of books in the home	0.263 **	0.145	0.602 **	0.558 **	1			
6. Composite index (SESR)	0.269 **	0.114	0.783 **	0.875 **	0.860 **	1		
7. Daily communication activities	0.085	−0.019	0.392 **	0.246 **	0.317 **	0.356 **	1	
8. Family literacy activities	0.217 *	0.025	0.234 **	0.241 **	0.296 **	0.306 **	0.474 **	1

Note: * *p* < 0.05. ** *p* < 0.01. NH: normal-hearing. SESR = Composite index of family socioeconomic resources. This composite variable combines measures of family economic level, parental education, and the number of books in the home.

**Table 7:** Hierarchical regression analysis predicting language abilities for preschool children with cochlear implants (n = 118).

		Unstandardized Coefficient		Standardized Coefficient	*t*	*p*	VIF	R^2^	Adjusted R^2^	△R^2^	F
Model		β	Standard Error	Beta							
1	(Constant)	−26.247	25.35		−1.035	0.303		0.232	0.218	0.232	17.334 **
	Age	19.73	5.46	0.30	3.61	<0.001	1.00				
	Composite index (SESR)	4.36	0.92	0.39	4.74	<0.001	1.00				
2	(Constant)	−3.263	24.52		−0.133	0.894		0.327	0.309	0.095	16.057 **
	Age	3.40	6.55	0.05	0.52	0.605	1.63				
	Composite index (SESR)	3.10	0.92	0.28	3.37	0.001	1.13				
	Duration of CI use	22.01	5.49	0.41	4.01	<0.001	1.75				
3	(Constant)	−30.522	27.11		−1.126	0.263		0.354	0.331	0.028	4.843 **
	Age	2.89	6.45	0.04	0.45	0.655	1.63				
	Composite index (SESR)	2.40	0.96	0.21	2.50	0.014	1.27				
	Duration of CI use	20.91	5.43	0.39	3.85	<0.001	1.76				
	Family literacy activities	3.18	1.44	0.18	2.20	0.030	1.19				
4	(Constant)	−61.385	27.52		−2.23	0.028		0.414	0.387	0.059	11.332
	Age	1.24	6.19	0.02	0.20	0.842	1.64				
	Composite index (SESR)	1.68	0.94	0.15	1.78	0.078	1.34				
	Duration of CI use	19.08	5.22	0.35	3.65	<0.001	1.78				
	Family literacy activities	0.70	1.57	0.04	0.45	0.655	1.52				
	Daily communication activities	4.65	1.38	0.31	3.37	0.001	1.63				

Note: ** *p* < 0.01. CI: cochlear implant. SESR = Composite index of family socioeconomic resources. This composite variable combines measures of family economic level, parental education, and the number of books in the home.

**Table 8:** Hierarchical regression analysis predicting language abilities for NH preschool children (n = 135).

		Unstandardized Coefficient		Standardized Coefficient	*t*	*p*	VIF	R^2^	Adjusted R^2^	△R^2^	F
Model		β	Standard Error	Beta							
1	(Constant)	30.50	9.95		3.07	0.003		0.598	0.592	0.598	98.288 **
	Age	28.95	2.20	0.73	13.15	<0.001	1.01				
	Composite index (SESR)	1.40	0.42	0.19	3.34	0.001	1.01				
2	(Constant)	23.01	15.51		1.48	0.140		0.599	0.590	0.001	0.398
	Age	29.04	2.21	0.73	13.13	<0.001	1.02				
	Composite index (SESR)	1.30	0.45	0.17	2.88	0.005	1.16				
	Daily communication activities	0.43	0.68	0.04	0.63	0.529	1.15				
3	(Constant)	18.85	15.21		1.24	0.217		0.622	0.610	0.022	7.597 **
	Age	28.94	2.16	0.73	13.41	<0.001	1.02				
	Composite index (SESR)	1.10	0.44	0.15	2.47	0.015	1.20				
	Daily communication activities	−0.393	0.73	−0.034	−0.542	0.589	1.38				
	Family literacy activities	1.88	0.68	0.17	2.76	0.007	1.33				

Note: ** *p* < 0.01. NH: normal-hearing. SESR = Composite index of family socioeconomic resources. This composite variable combines measures of family economic level, parental education, and the number of books in the home.

## Data Availability

The datasets used or analyzed in this study are available from the corresponding author upon request.

## Supplementary Materials

The following supporting information can be downloaded at: https://www.mdpi.com/article/10.3390/audiolres16010018/s1, File S1: Survey questionnaire on the current status of language activities in the family life of preschool children with cochlear implants

## Abbreviations

The following abbreviations are used in this manuscript:

CIs	Cochlear implants
NH	Normal hearing
SES	Socioeconomic status
SESR	Socioeconomic status resource
ASQ	Ages Stages Questionnaire
MCELP-CS	Mandarin Clinical Evaluation of Language for Preschoolers’ Core Scale
