# Supplementary material for: Home Language Activities and Language Ability Between Chinese Preschool Children with Cochlear Implants and Children with Normal Hearing
Source: Audiol Res. 2026 Jan 28;16(1):18. doi: 10.3390/audiolres16010018 (PMC12921801; doi:10.3390/audiolres16010018)
Supplement: Supplementary file 1 [file audiolres-16-00018-s001.zip › audiolres-4056731-supplementary.pdf]

Supplementary Material File S1

Survey questionnaire on the current status of language activities in the family life of preschool children with cochlear implants

I . Basic information of preschoolers with cochlear implants

1. Age:______ years_____ months
2. Gender: 1) Male 2) Female
3. Age at hearing loss diagnosis:______ years______ months
4. Age at cochlear implant initiation: ______years______ months

5. Cochlear implant status
   1) Bilateral 2) Right Ear 3) Left Ear
   If implanted in only one ear, does the other ear use a hearing aid?
   1)Yes 2) No

6. Hearing levels
   ① Average pre-implant hearing (Unassisted)
   1) Right ear_________dB HL 2) Left ear_________dB HL
   ② Average hearing level after cochlear implantation or hearing aid use
   1) Right ear_________dB HL 2) Left ear_________dB HL
7. Does your child have any other disabilities besides hearing impairment?
   1) Yes 2) No

8. Educational attainment of your child's parents
   ① Father's educational attainment
   1) Junior high school or below 2) High school 3) College diploma
   4) Bachelor's degree 5) Master's degree or above
   ② Mother's educational attainment
   1) Junior high school or below 2) High school 3) College diploma
   4) Bachelor's degree 5) Master's degree or above

9. Your household's economic level (annual income)
   1) 0-30,000 yuan 2) 30,000-80,000 yuan 3) 80,000-150,000 yuan
   4) 150,000-300,000 yuan 5) 300,000 yuan and above

10. How many books do your children own at home?
    1) Fewer than 10 books 2) 11-30 books 3) 31-60 books 4) 61-100 books 5) More than 100 books

11. How many books do the adults in your household own?
    1) Less than 10 books 2) 11-30 books 3) 31-60 books 4) 61-100 books 5) Over 100 books

Ⅱ. Language activities in everyday family life

NO.	Item	Rarely	Occasionally	Sometimes	Often	Always
1	Frequency of communication between the primary caregiver and your child(e.g., discussing experiences the child has had, or shared experiences and mutual interests between the caregiver and the child.)	1	2	3	4	5
2	Frequency of communication between your child and their siblings or other children (e.g., discussing experiences the child has had personally, or shared experiences and mutual interests with other children.	1	2	3	4	5
3	Frequency of your child initiates conversations by asking questions or telling stories about their day to family members.	1	2	3	4	5
4	Frequency of your child's independent reading.	1	2	3	4	5
5	Frequency of shared picture book reading activities between the primary caregiver and your child.	1	2	3	4	5
6	Frequency of the primary caregiver and child engage in joint activities at home (e.g., singing, drawing, Doing handicrafts,doing housework) aside from book reading.	1	2	3	4	5
7	Frequency of the primary caregiver takes the child out for play or excursions	1	2	3	4	5
8	Frequency of your child's play with siblings or other children.	1	2	3	4	5
9	Frequency of child engages in other language-learning activities at home (e.g., nursery rhymes, math, ancient poems, pinyin), excluding picture book reading.	1	2	3	4	5
